# Non-fungal pathogens detected by broad-range fungal polymerase chain reaction

**DOI:** 10.1128/jcm.00087-25

**Published:** 2025-06-10

**Authors:** Sonya Ahuja, Joshua A. Lieberman

**Affiliations:** 1 Department of Laboratory Medicine and Pathology, University of Washington School of Medicine 189576, Seattle, Washington, USA; University of Calgary, Calgary, Alberta, Canada

**Keywords:** fungal PCR, parasitology, 28S rRNA sequencing, ITS rRNA sequencing, molecular diagnostics, neglected tropical diseases

## LETTER

Few laboratory tests target non-fungal eukaryotic pathogens, such as oomycetes, amoebae, and other parasites. At our molecular reference laboratory, we observed rare detections (<1%) of non-fungal eukaryotic pathogens by broad-range fungal PCR and Sanger sequencing (1–3) due to conserved primer binding sequences in the large ribosomal subunit gene (Fig. S1) (4). These were reported clinically but described as “incidental findings” whose presence could preclude the detection of fungal DNA (see Supplemental materials). To assess the diversity and taxonomic specificity of organisms detected by this method, we searched the laboratory information system for the term “incidental” within broad-range fungal PCR reports from January 2001 to August 2023. For organisms identified by this strategy, we searched fungal PCR reports for instances where “incidental” was omitted. Metadata and laboratory results were analyzed using R version 2023.06.2+561.

**Fig 1:**
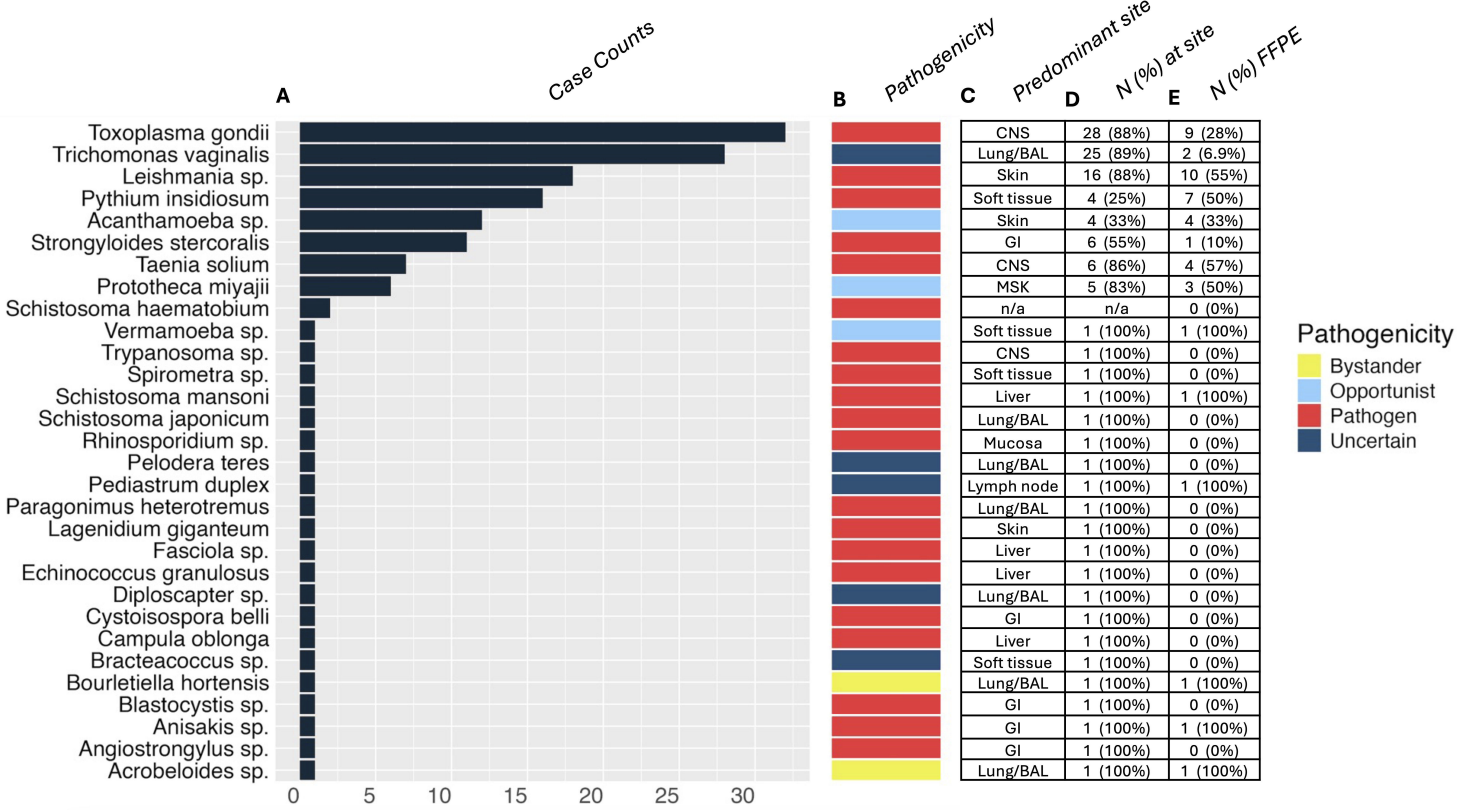
Characteristics of incidentally identified non-fungal eukaryotes**.** Case counts of non-fungal organisms detected are displayed as horizontal bars (**A**). Note that *Campula oblonga* was detected from a dolphin necropsy performed for research testing rather than from a human case. Organism pathogenicity is indicated by colored bars (**B**). The anatomic site where the incidental organism was most often detected is indicated (GI, gastrointestinal; BAL, bronchoalveolar lavage; and n/a, not applicable) (**C**), with the proportion of cases at the predominant site provided as the number and percentage of cases (**D**). The number and percentage of cases for each organism detected in formalin-fixed paraffin-embedded tissue were calculated using all samples tested rather than unique patients (**E**).

We identified 153 cases from 138 patients, with a median age of 49.6 years. Of the patients, 50 were female, 86 were male, and 2 were unspecified. Thirty unique non-fungal organisms distributed across 10 taxonomic orders were reported (Fig. S2). Of eight identified in multiple patients, seven were *bona fide* pathogens (Fig. 1). The three most common were *Toxoplasma gondii* (*N* = 32), *Trichomonas vaginalis* (*N* = 28), and *Leishmania* sp. (*N* = 18) (Fig. 1). *T. gondii* was primarily identified in central nervous system (CNS) samples (28/32), and *Leishmania* sp. was primarily in skin (16/18). Interestingly, *T. vaginalis* was primarily identified in BAL/lung specimens (25/28). Most organisms were detected in viscera (*N* = 68), with BAL/lung (*N* = 37) as the most frequently involved site. The 28S primer set was more frequently positive (114/167) than ITS (66/167), likely reflecting increased sequence diversity in ITS loci (Fig. S1) (4). Both primer sets were positive in 37 cases.

We identified a wide range of non-fungal incidental findings, from the common helminth *Strongyloides stercoralis* (*N* = 14) to uncommon pathogens like *Pythium insidiosum* (*N* = 20). Five organisms were of uncertain clinical significance, each identified in one case (Fig. 1). While some may have been contaminants, others, like *Bracteacoccus*—a green alga identified in subcutaneous tissue from a patient’s thumb—could represent true infections. Our observations suggest broad-range fungal assays may aid in the detection and identification of non-fungal eukaryotic pathogens when other diagnostic options are limited, including when present in formalin-fixed paraffin-embedded tissue (Fig. 1).

This study has several limitations. First, the assay was validated for fungi; thus, performance data for other organisms are lacking. Second, concurrent histologic/laboratory results were frequently unavailable, limiting clinicopathologic correlation and precluding an estimate of false negativity (see Supplemental materials). Third, the assay was frequently limited to genus rank identification due to inherent limitations in the targeted rRNA loci or lack of high-quality sequences in public databases (5). Consequently, less common parasites, such as *Spirometra* and *Angiostrongylus*, were reported at genus rank, whereas more common parasites like *Strongyloides stercoralis* and *Schistosoma* spp. were identified at species rank. Notably, 28S rRNA sequencing of *Leishmania* spp. was limited to genus identification; however, species identification informs clinical decisions (6), underscoring the importance of organism-specific assays (7). Despite these limitations, our results indicate that fungal PCR may prove a valuable adjunct in the detection of rare eukaryotic pathogens when suspected by visualization in histologic sections or from serologic testing and clinical presentation.

## Data Availability

Representative 28S sequences are available as GenBank accessions PV682825 through PV682886.

## SUPPLEMENTAL MATERIAL

Figure S1 jcm.00087-25-s0001.tiff Multisequence alignment of primer binding sites.

Figure S2 jcm.00087-25-s0002.tiff Phylogenetic tree with representative 28S sequences.

Supplemental material jcm.00087-25-s0003.docx Supplemental document includes additional details on laboratory methods - wet bench, bioinformatics, and reporting methods - as well as primer binding site sequence analysis and comments on clinical significance & assay validation.

Table S1 jcm.00087-25-s0004.xlsx Table of sequences included for primer binding site MSA.

ASM does not own the copyrights to Supplemental Material that may be linked to, or accessed through, an article. The authors have granted ASM a non-exclusive, world-wide license to publish the Supplemental Material files. Please contact the corresponding author directly for reuse.
