# Supplementary material for: Non-fungal pathogens detected by broad-range fungal polymerase chain reaction
Source: J Clin Microbiol. 2025 Jun 10;63(7):e00087-25. doi: 10.1128/jcm.00087-25 (PMC12239715; doi:10.1128/jcm.00087-25)
Supplement: Supplemental material — Supplemental document includes additional details on laboratory methods - wet bench, bioinformatics, and reporting methods - as well as primer binding site sequence analysis and comments on clinical significance & assay validation. [file jcm.00087-25-s0003.docx]

**Title**: Non-fungal Pathogens Detected by Broad-Range Fungal Polymerase Chain Reaction

**Authors**: Sonya Ahuja^1^, Joshua A. Lieberman^1#^

**Affiliations**: ^1^Department of Laboratory Medicine and Pathology, University of Washington School of Medicine, Seattle, Washington, USA

**#Corresponding Author:** Joshua A. Lieberman, joshuaal@uw.edu, Department of Laboratory Medicine and Pathology, Box 357110, 1959 NE Pacific St, Seattle, WA 98195‐7110

**Supplemental Materials**

*Supplemental Methods.*

*Laboratory Procedures and Accepted Specimen Types*

The laboratory accepted non-blood body fluids, fresh tissue, formalin-fixed paraffin embedded tissue, and swabs for broad-range fungal PCR clinical testing. Clinical metadata, including histopathology slides or images, were not required but occasionally received by the laboratory; such data were available for in-house cases. DNA was extracted from each sample type following methods validated in the laboratory for each matrix type (1).

Following DNA extraction, fungal PCR amplicons were prepared using the BigDye Xterminator reaction kit (ThermoFisher Scientific) prior to bidirectional Sanger sequencing and multiple layers of case review. Cases were first reviewed by two medical laboratory scientist staff certified by the American Society for Clinical Pathology and then by a laboratory director (1). Obtained sequences were trimmed to remove low quality bases (Phred score < 20) at the 5’ and 3’ termini and to remove primer sequences, then evaluated by Basic Localized Search Tool (BLAST) (2) against both public databases in NCBI and a curated, in-house database that represents both public sequences from GenBank and sequences from clinical testing (1). The laboratory’s procedures for evaluating sequences and reporting non-fungal pathogens did not change significantly during the study period.

*Bioinformatic Analysis*

Species-rank identification required ~99.5% nucleotide identity to established in-house sequence records or GenBank refseq records, from type material, and/or published in a peer-reviewed manuscript. Genus-rank identification required >97% nucleotide identity to records from the databases. When BLAST results met species-rank reporting criteria for multiple species within a genus, results were reported at genus-rank.

Representative sequences obtained were MAFFT aligned in Unipro Ugene (ver 44.0) (3). Trees were generated by IQ-Tree with automatic substitution model optimization (4, 5) and visualized with ggtree (6).

*Reporting and Retrospective Case Identification*

Incidental findings were reported clinically. In the clinical result, non-fungal identifications were preceded by the phrase “Incidental Finding” to distinguish from fungal targets for which the assay was validated. Searching the laboratory information system (LIS) for “incidental” this identified most cases. This laboratory protocol did not change over the course of the study period. However, we were aware of more cases for several organisms (e.g., *Schistosoma hematobium*, *Bracteococcus sp*) that were missed by this case finding strategy, either because the “incidental finding” phrase was not added or, in the case of *Bracteococcus sp*, the report was initially reported as “indeterminate due to interfering substances” and then amended to report the algae. Therefore, we also performed a manual review of records in the middleware for each genus identified in the LIS search.

*Supplemental Results*.

To compare sequences of primer binding sites for the 28S (NL1/NL4), ITS1 (ITS1/ITS2), and ITS2 primers (ITS3/ITS4; ITS3 was not analyzed separately as it is the reverse complement of ITS2) we performed BLAST analyses using the core_nr or rRNA (28S/ITS) GenBank databases but limited to each organism identified in this study. We extracted organism-specific primer binding site sequences in the rRNA gene targets and corresponding GenBank accessions for representative sequences (**Table S1**). We represented discordance between GenBank records with single letter IUPAC representations of the corresponding ambiguous in a multisequence MAFFT alignment of primer binding sites in available target sequences (**Figure S1)**.

Primer binding sites for 28S (NL1, NL4) were generally well-conserved across detected organisms. Common point mutations were in the 5’ end of primers with recurrent variation at +7 and +9 positions (**Figure S1**). Variation in primer binding site was observed for the reverse primer for the ITS1 locus (primer name ITS2, **Figure S1**), with less variation in the 5’ end. The ITS3 primer is reverse complementary to ITS2 and serves as the forward primer for the ITS2 locus. Both the 3’ ends of ITS3 and ITS4 are relatively conserved, with recurrent mutations observed at positions +16 and +18 of the ITS4 primer (**Figure S1**).

To compare sequence relatedness, we generated a tree of representative 28S sequences obtained from clinical samples (**Figure S2**). The sequenced loci are diverse and span more than a dozen Families. There was minimial sequence variation within genera of important parasitic pathogens, particularly *Acanthamoeba spp.* and *Leishmania* *spp* (**Figure S2**). Even across the two subgenera *Leishmania Leishmania spp.* and *Leishmania Viannia spp* we observed minimal sequence variation (**Figure S2**)

*Supplemental Discussion.*

Previous studies have indicated that broad-range fungal PCR is capable of detecting non-fungal pathogens. However, reports have been limited to case studies reporting detection of important parasitic pathogens such as *Strongyloides stercoralis* (7), *Cystoisospora belli* (8), *Taenia solium* (9), the uncommon oomycete pathogen *Pythium insidiosum* (10), and embedded in studies of the performance of broad-range fungal PCR reporting rare detection of *Toxoplasma gondii*, *Trypanosoma cruzi*, and *Leishmania* spp. (11). Here, we demonstrate that broad-range fungal PCR targeting the large rRNA gene and Internal Transcribed Spacer (ITS) loci can detect a broad array of non-fungal pathogens (**Figure S2**). Identification is possible due to high percent identity, 85-100%, of broad-range fungal primers to such “off-target” organism genomic targets with polymorphisms primarily located in the 5’ end of primers (**Figure S1**).

These findings raise three key questions: 1) how to report these results; 2) how to integrate with other laboratory studies, including histology findings, to drive patient care; and 3) how to incorporate non-fungal pathogens into validation studies. Our laboratory’s policy is to always report well-established parasite pathogens provided quality control metrics are acceptable. However, these results are labeled in the clinical report as “incidental findings” to indicate the analytical performance of the test for these organisms is not known since they were not included in the validation study (12). Additionally, we comment that the presence of such off-target DNA means we cannot rule out the presence of fungal organisms. Given the variation in nucleotide identity between primers and primer binding sites we do not require both 28S and ITS amplicons to be positive to report a parasite by fungal PCR.

Integrating these results with other laboratory tests is important, particularly since FFPE tissue may be falsely negative or falsely detect contaminants (13). Most cases we detected were submitted through the reference laboratory and thus histopathology findings were not available to us. Nonetheless, some case have corresponding histopathology that is concordant with the molecular detection (7–10), consistent with the observation that the yield of fungal PCR in tissue is highest when organisms are observed (14, 15)*.* The pathogens reported in this study (**Figure 1**) have not been encountered as contaminants in our laboratory’s historical testing, are uncommon in the United States and thus unlikely to be reagent contamination. Thus, fungal PCR is most likely useful for parasites when they are observed in tissue. *Pythium* in particular can mimic fungal morphology (10). We suspect that identification of established pathogens like *Fasciola*, *Leishmania*, *Pythium*, *Rhinosporidium, Trypanosoma*, *Strongyloides, Schistosoma,* and *Prototheca* are almost always clinically relevant.

More challenging is the decision to report potential bystander organisms, such as insects (e.g., *Bourletiella hortensis*) or nematodes of uncertain or unlikely clinical significance (e.g., *Diploscapter sp.*). In general, we have elected to report when quality metrics are acceptable, including robust amplification and sequence quality, and the detected sequences have not been encountered by our laboratory as would be expected with reagent background. We include the same comments indicating an “incidental finding” that precludes detection of fungal organisms. Although other laboratories may establish different policies, we err on the side of reporting to permit case detection with rare or emerging pathogens or, at a minimum, to indicate the source of interfering DNA. In such cases, correlation with histopathology is essential.

In the future, it may be useful to include parasites in the validation of fungal PCR. However, given the high percent identity of fungal rRNA primers to target sequences in parasites (**Figure S1**) it may not be possible to design fungal PCR assays that remain broadly inclusive to fungi but exclude parasite detection. Additionally, obtaining sufficient organisms or parasite DNA for confident determination of analytical sensitivity may be difficult. Laboratories performing fungal PCR assays will need to balance the benefits of knowing these performance characteristics with the costs of extending their validation to include diverse organisms that may only be encountered a few times per decade.

**Figure Legends**

**Supplemental Figure S1. Multisequence alignment of broad-range fungal primers and primer binding sites.** Primer sequences were compared to the rRNA sequences of detected non-fungal organisms identified in this study. The 28S rRNA locus was amplified with NL1 and NL4 primers. The ITS1 locus was amplified with ITS1 and ITS2 primers. The ITS2 locus was amplified with ITS3 and ITS4 primers. Note that ITS3 is the reverse complement of ITS2 and results are presented using the ITS2 sequence. Dashes indicate sequence was not available on core_nt databases in Genbank. IUPAC codes for wobble bases were included when multiple rRNA alleles for a target organism were present in Genbank.

**Supplemental Figure S2. Phylogenetic relationships among representative sequences obtained from non-fungal organisms in clinical testing.** 28S sequences obtained from clinical sequence were aligned, used to generate a treefile, and visualized in ggtree. Genetic distance is represented along the x-axis and expressed as substitutions per site. Branch tips are in purple with corresponding organism names connected by dotted lines. The tree is annotated with color-coded taxonomic assignments for Class, Order, and Family to represent the sequence diversity.

*Supplemental References*

1. Lieberman JA, Bryan A, Mays JA, Stephens K, Kurosawa K, Mathias PC, SenGupta D, Bourassa L, Salipante SJ, Cookson BT. 2021. High Clinical Impact of Broad-Range Fungal PCR in Suspected Fungal Sinusitis. J Clin Microbiol JCM0095521.

2. Johnson M, Zaretskaya I, Raytselis Y, Merezhuk Y, McGinnis S, Madden TL. 2008. NCBI BLAST: a better web interface. Nucleic Acids Res 36:W5-9.

3. Okonechnikov K, Golosova O, Fursov M, UGENE team. 2012. Unipro UGENE: a unified bioinformatics toolkit. Bioinformatics 28:1166–1167.

4. Minh BQ, Schmidt HA, Chernomor O, Schrempf D, Woodhams MD, von Haeseler A, Lanfear R. 2020. IQ-TREE 2: New Models and Efficient Methods for Phylogenetic Inference in the Genomic Era. Mol Biol Evol 37:1530–1534.

5. Trifinopoulos J, Nguyen L-T, von Haeseler A, Minh BQ. 2016. W-IQ-TREE: a fast online phylogenetic tool for maximum likelihood analysis. Nucleic Acids Res 44:W232-235.

6. Yu G. 2020. Using ggtree to Visualize Data on Tree-Like Structures. Current Protocols in Bioinformatics 69:e96.

7. Konnick EQ, Chow S-K, Reder NP, Sengupta DJ, Hoogestraat DR, Pottinger PS, Abbott AN, Monsaas PW, Kurosawa K, Stephens K, Salipante SJ, Yeung CCS. 2016. Incidental identification of Strongyloides stercoralis infection by broad-range 28S rDNA gene sequencing in a patient with a hematolymphoid malignancy. Diagn Microbiol Infect Dis 86:362–364.

8. Murphy SC, Hoogestraat DR, Sengupta DJ, Prentice J, Chakrapani A, Cookson BT. 2011. Molecular diagnosis of cystoisosporiasis using extended-range PCR screening. J Mol Diagn 13:359–362.

9. Harrington AT, Creutzfeldt CJ, Sengupta DJ, Hoogestraat DR, Zunt JR, Cookson BT. 2009. Diagnosis of neurocysticercosis by detection of Taenia solium DNA using a global DNA screening platform. Clin Infect Dis 48:86–90.

10. Salipante SJ, Hoogestraat DR, SenGupta DJ, Murphey D, Panayides K, Hamilton E, Castañeda-Sánchez I, Kennedy J, Monsaas PW, Mendoza L, Stephens K, Dunn JJ, Cookson BT. 2012. Molecular diagnosis of subcutaneous Pythium insidiosum infection by use of PCR screening and DNA sequencing. J Clin Microbiol 50:1480–1483.

11. Gomez CA, Budvytiene I, Zemek AJ, Banaei N. 2017. Performance of Targeted Fungal Sequencing for Culture-Independent Diagnosis of Invasive Fungal Disease. Clin Infect Dis 65:2035–2041.

12. Rakeman JL, Bui U, Lafe K, Chen Y-C, Honeycutt RJ, Cookson BT. 2005. Multilocus DNA sequence comparisons rapidly identify pathogenic molds. J Clin Microbiol 43:3324–3333.

13. Stempak LM, Vogel SA, Richter SS, Wyllie R, Procop GW. 2019. Routine Broad-Range Fungal Polymerase Chain Reaction With DNA Sequencing in Patients With Suspected Mycoses Does Not Add Value and Is Not Cost-Effective. Arch Pathol Lab Med 143:634–638.

14. Clark ST, Yau YCW, Campigotto A, Gharabaghi F, Richardson SE, Tadros M. 2022. Assessment of panfungal PCR performance with formalin-fixed paraffin-embedded tissue specimens†. Med Mycol 60:myac004.

15. Sparks R, Halliday CL, Green W, Chen SC-A. 2023. Panfungal PCR on formalin-fixed, paraffin-embedded tissue: to proceed or not proceed? Pathology 55:669–672.
